# Dietary factors associated with obesity indicators and level of sports participation in Flemish adults: a cross-sectional study

**DOI:** 10.1186/1475-2891-6-26

**Published:** 2007-09-21

**Authors:** Nathalie Duvigneaud, Katrien Wijndaele, Lynn Matton, Renaat Philippaerts, Johan Lefevre, Martine Thomis, Christophe Delecluse, William Duquet

**Affiliations:** 1 Department of Human Biometry and Biomechanics, Faculty of Physical Education and Physical Therapy, Vrije Universiteit Brussel, Pleinlaan 2, B-1050 Brussel, Belgium; 2 Department of Movement and Sports Sciences, Ghent University, Watersportlaan 2, B-9000 Gent, Belgium; 3 Department of Biomedical Kinesiology, Faculty of Kinesiology and Rehabilitation Sciences, K.U.Leuven, Tervuursevest 101, B-3001 Leuven, Belgium

## Abstract

**Background:**

Obesity develops when energy intake continuously exceeds energy expenditure, causing a fundamental chronic energy imbalance. Societal and behavioural changes over the last decades are held responsible for the considerable increase in sedentary lifestyles and inappropriate dietary patterns. The role of dietary fat and other dietary factors in the aetiology and maintenance of excess weight is controversial. The purposes of the present study were to investigate the dietary factors associated with body mass index (BMI) and waist circumference (WC), and to analyse whether dietary intake varies between subjects with different levels of sports participation.

**Methods:**

Data for this cross-sectional study, including anthropometric measurements, 3-day diet diary and physical activity questionnaire, were collected by the Flemish Policy Research Centre Sport, Physical Activity and Health (SPAH) between October 2002 and April 2004. Results of 485 adult men and 362 women with plausible dietary records were analysed. Analyses of covariance were performed to determine the differences in dietary intake between normal weight, overweight and obese subjects, and between subjects with different levels of sports participation.

**Results:**

Total energy intake, protein and fat intake (kcal/day) were significantly higher in obese subjects compared to their lean counterparts in both genders. Percentage of energy intake from fat was significantly higher in obese men compared to men with normal weight or WC. Energy percentages from carbohydrates and fibres were negatively related to BMI and WC in men, whereas in women a higher carbohydrate and fibre intake was positively associated with obesity. Alcohol intake was positively associated with WC in men. Subjects participating in health related sports reported higher intake of carbohydrates, but lower intake of fat compared to subjects not participating in sports.

**Conclusion:**

This study supports the evidence that carbohydrate, fat, protein and fibre intake are closely related to BMI and WC. The sex differences for dietary intake between obese men and women might reflect the generally higher health consciousness of women. Alcohol intake was only associated with WC, emphasizing the importance of WC as an additional indicator in epidemiological studies. Besides enhancing sports and physical activity, it is necessary to improve the knowledge about nutrition and to promote the well-balanced consumption of wholesome food.

## Background

Obesity is a worldwide escalating problem caused by a complex interaction of genetic, socio-demographic, behavioural and environmental factors. There is large evidence that obesity develops when energy intake continuously exceeds energy expenditure, causing a fundamental chronic energy imbalance. Societal and behavioural changes over the last decades are held responsible for the considerable increase in sedentary lifestyles [1], as well as inappropriate dietary patterns including snacking, large portion sizes, soft drinks, high fat and energy dense diets [2]. The role of high dietary fat intake in the aetiology and maintenance of excess weight is controversial. Positive associations between dietary fat and excess body fat were observed in some studies, but not in others [3]. Other dietary factors besides dietary fat are now considered to influence obesity i.e. carbohydrate, protein, fibre, energy density and glycemic index [4-6]. A better understanding of these factors is essential to innovate more appropriate health policies.

The relative importance of dietary intake in the development of obesity is difficult to establish because of dietary reporting bias. In general, inaccurate energy intake reports result from underrecording, undereating during the period of dietary registration or a combination of both [7]. Overrecording may also occur, though infrequent [7]. Studies identifying low energy reporters and factors associated with underreporting used the doubly labelled water technique [8,9] or the ratio of energy intake to basal metabolic rate [10,11]. It has been observed that overweight and obese individuals tend to underreport their dietary intake to a greater extent than normal weight individuals [12-14]. This phenomenon may lead to the paradoxal observation that obese individuals appear to eat less than lean individuals. Sex, age, smoking, physical activity level, educational level, body image, health consciousness and social desirability are other factors reported to affect the accuracy of self-reported dietary intake [13-15].

Some studies have examined the association of nutritional intake with BMI [4] and more specifically with overweight and obesity [16-18]. However, for a number of populations, including the Flemish, information on this relationship is missing. Also, although the associations of abdominal obesity with type 2 diabetes, cardiovascular disease [19] and mortality appear to be stronger than for general obesity, only few studies have published results describing the relationship between dietary intake and waist circumference in a cross-sectional [20-22] or prospective design [23-26]. Moreover, dietary intake was not always the main focus in these studies.

The first purpose of the present study was to analyse differences between plausible reporters and underreporters in a sample of Flemish adults. A second purpose was to investigate the associations of dietary intake with both BMI and WC, covaried for age and physical activity variables. A third purpose was to analyse whether dietary intake varies between subjects with different levels of sports participation, with age and BMI as covariates.

## Methods

### Subjects

Data used in the present study were collected by the Flemish Policy Research Centre Sport, Physical Activity and Health (SPAH) between October 2002 and April 2004. One of the aims of the SPAH study was to investigate the cross-sectional relationship between physical activity, physical fitness and several health parameters among the adult population of Flanders, the Flemish part of Belgium. For this purpose, 46 Flemish municipalities were selected on the basis of clustered random sampling. Within these municipalities, the National Institute of Statistics randomly selected a sample of Flemish men and women aged 18 to 75 years. In each municipality, the size of the random sample was proportionate to its population size. Detailed establishment and description of this sample have been given elsewhere [27]. A total of 5170 subjects volunteered to participate in the SPAH Study. This sample was compared with the total Flemish adult population to evaluate its representativeness. Although some small differences were observed, the sample of the SPAH Study can be considered as sufficiently representative for geographic distribution, age, gender and educational level [27]. Only 1520 subjects filled in a 3-day diet record and could be included in the present analyses. Consequently, we can not assume that they are fully representative for the Flemish adult population. Participants with incomplete data for anthropometry and/or physical activity were also excluded, as well as participants with implausible energy intake. All subjects signed an informed consent statement and received explanation about the purpose and procedures of the study before participating. The study was approved by the ethical and medical committee of the Katholieke Universiteit Leuven.

### Dietary assessment

The subjects completed a validated 3-day diet record, the three days being two weekdays and one weekend day [28]. Whenever possible the subjects were instructed to weigh the amount of foods eaten. Nevertheless, if weighing was not possible, the subjects were instructed to estimate the amount of all foods eaten, using standard household measures, e.g. tablespoon (15 g). This information was included in the 3-day diet record booklet. This method aimed to increase the quality of the food estimation. Diet records were analysed using Becel Nutrition software (Unilever Co.; Rotterdam, the Netherlands) based on the Belgian (NUBEL 1999) and Dutch (NEVO 2001) food composition data banks. The dietary factors reported were total energy intake (kcal/day), protein, carbohydrates (sugar, starch), fibre, fats (saturated, monounsaturated, polyunsaturated), alcohol, calcium (mg/day), iron (mg/day) and cholesterol. Macronutrients, fibre and alcohol intake were expressed in kcal per day (kcal/day) and as percentages of total energy intake (%).

### Evaluation of inaccurate reports of dietary intake

The method for screening out inaccurate reports of dietary energy intake proposed by McCrory and colleagues [7] was used in the present study. Physiologically implausible energy intake reports were identified by comparing reported energy intake (rEI) with predicted total energy expenditure (pTEE). The equation of Vinken et al. [29] was used to predict TEE in each subject. This equation to predict adult energy requirements from simple anthropometric and laboratory measures was developed by using the doubly labeled water method [29] and is at follows:

pTEE = 7.377 - 0.073 × age + 0.0806 × weight + 0.0135 × height - 1.363 × sex

where age is in years, weight in kg, height in cm, and sex is 0 for men and 1 for women. Based on the principles outlined by Black [10], specific cut-offs for reported energy intake as percentage of predicted total energy expenditure were calculated, taking into account technical error of measuring energy expenditure by the doubly labelled water method (CV_tmTEE_), biological intraindividual variation in reported energy intake (CV_wEI_) and predicted total energy expenditure (CV_wpTEE_). For the present analysis, the ± 1 SD cut-off was chosen above the ± 2 SD cut-off to increase the validity of reported energy intake. It has also been suggested that the use of ± 1 SD cut-off may be preferable when examining relationships between diet and health [7], which is the case in this study. The SD cut-off formula used was:

±1 SD=(CVwEI2/d)+CVwpTEE2+CVtmTEE2
 MathType@MTEF@5@5@+=feaafiart1ev1aaatCvAUfKttLearuWrP9MDH5MBPbIqV92AaeXatLxBI9gBaebbnrfifHhDYfgasaacH8akY=wiFfYdH8Gipec8Eeeu0xXdbba9frFj0=OqFfea0dXdd9vqai=hGuQ8kuc9pgc9s8qqaq=dirpe0xb9q8qiLsFr0=vr0=vr0dc8meaabaqaciaacaGaaeqabaqabeGadaaakeaacqGHXcqScqaIXaqmcqqGGaaicqWGtbWucqWGebarcqGH9aqpdaGcaaqaaiabcIcaOiabdoeadjabdAfawnaaDaaaleaacqWG3bWDcqWGfbqrcqWGjbqsaeaacqaIYaGmaaGccqGGVaWlcqWGKbazcqGGPaqkcqGHRaWkcqWGdbWqcqWGwbGvdaqhaaWcbaGaem4DaCNaemiCaaNaemivaqLaemyrauKaemyraueabaGaeGOmaidaaOGaey4kaSIaem4qamKaemOvay1aa0baaSqaaiabdsha0jabd2gaTjabdsfaujabdweafjabdweafbqaaiabikdaYaaaaeqaaaaa@5398@

with CV_wEI _= 23%; d = 3 (3 days of diet record); CV_wpTEE _= 17.7%; CV_tmTEE _= 8.2%

When using the formula above, the ± 1 SD for the agreement between rEI and pTEE is approximately ± 24%. This means that an individual's rEI must be between 76% and 124% of pTEE to be considered as physiologically plausible. About 52% of the total sample was identified as plausible reporters (485 men; mean age = 50.2 ± 13.3 years and 362 women; mean age = 51.4 ± 12.9 years), 41.2% of the male (n = 382) and 42.2% (n = 291) of the female subjects were detected as underreporters. The overreporters represented only a small part of the total sample (men: 6.6%; women: 5.4%).

### Anthropometric measurements

Anthropometric measurements were performed by trained staff using standardized procedures and equipment as proposed by the International Society for the Advancement of Kinanthropometry [30]. Measurements were taken with participants barefoot and in minimal clothing. Body weight was recorded to the nearest 0.1 kg with a digital weight scale (Seca 841, Seca GmbH, Hamburg, Germany) and body height with a Holtain stadiometer (Holtain, Crymych, UK) to the nearest mm. Waist circumference was measured using a metal tape (Rosscraft, Surrey, BC, Canada) to the nearest mm, at the narrowest level between the most inferior rib margin and the iliac crest. Skinfold thicknesses (biceps, triceps, subscapular, supra-iliac, thigh and medial calf) were measured twice on the right side of the body using a Harpenden calliper (Holtain, Crymych, UK). The sum of these six skinfold thicknesses was calculated.

### Physical activity assessment

Educational level and some physical activity variables were evaluated using the Flemish Physical Activity Computerized Questionnaire (FPACQ). Participants were classified into low (primary school), moderate (secondary school) and high (college or university) educational levels. The FPACQ was found to be a reliable and valid questionnaire for the assessment of different dimensions of physical activity during a usual week in employed/unemployed and retired people [31]. For the present study, three variables of physical activity were calculated: time spent in health related sports (Tsport), time spent watching TV or using the computer (Ttv) and physical activity level (PAL). Tsport was evaluated by asking respondents to select their three most important sports activities from a list of 196 sports. For each of these sports activities, frequency (from once/year to more than once/day) and duration (from some h/year to more than 20 h/week) were also reported. For scaled classification of exercise intensity, the MET-value of each sports activity was determined according to Ainsworth et al. [32]. Dependent on age, the sports activities have to meet a minimal MET-value to induce health benefits. Therefore, the American College of Sports Medicine (ACSM) recommendations were used to determine which sports activities (h/week) could be considered as health related according to their MET-value [33]. For individuals younger than 35 years, the sport activities should have a MET-value ≥4.5. For individuals between 35 and 50 years, a MET-value ≥4 is necessary and for individuals of 50 years and older a MET-value ≥3.5 is sufficient to induce health benefits. Ttv (h/week) was calculated as an indicator of in-active or sedentary behaviour. Participants were asked to indicate time spent watching television or playing computer or video games during an average week day and weekend day (from 0 to ≥6 h/day). PAL (MET) is an indicator of the general activity level. To calculate PAL, the overall energy expenditure during a usual week was divided by 168 (number of hours in one week) and by body weight. The total energy expenditure is the sum of the energy expenditure of active leisure time physical activities, occupation and the energy expenditure related to eating, sleeping and the remaining quiet leisure time.

Smoking habit was assessed using the Monica Smoking Questionnaire [34]. According to their responses, the participants were classified into 3 groups: never, former and current smokers.

### Statistical analysis

Descriptive statistics (mean ± standard deviation or percentages) of plausible and underreporters were calculated. Independent *t *tests and chi-square tests were used to compare anthropometric, behavioural and dietary characteristics between plausible and underreporters. All analyses were conducted in both genders separately. To assess the associations between dietary intake and obesity, analyses were conducted in the total and plausible sample, but as it is commonly known that self-reported energy intake gives rise to a number of implausible reports that may lead to erroneous conclusions, the results were only presented for the plausible sample.

Analyses of covariance (ANCOVA) were performed to determine the mean differences in dietary intake among normal weight (BMI < 25 kg/m^2^), overweight (BMI 25–29.9 kg/m^2^) and obese subjects (BMI ≥30 kg/m^2^) with statistical control for age, Tsport and Ttv. These analyses were repeated to evaluate differences in dietary intake between obesity groups based on the WC classification proposed by the World Health Organization (WHO) [35]. WC values <94 cm in men and <80 cm in women are considered as normal with low health risk. Male subjects with WC between 94–102 cm and female subjects with WC between 80–88 cm are regarded to have moderate risk abdominal obesity. Men and women with, respectively, WC values ≥102 cm and ≥88 cm, are considered to have high risk abdominal obesity.

To assess differences in dietary intake among subjects with different levels of sports participation ANCOVA's were conducted between subjects with no sports participation, moderate and high sports participation with age and BMI as covariates. The physical activity recommendation, 30 min of moderate intensity activity/day, was used for classification in groups with different level of sports participation. Consequently, subjects with Tsports = 0 h/week were considered as sedentary, those with Tsports > 0 h/week but < 3.5 h/week as moderately active and those with Tsports ≥3.5 h/week as highly active [36]. All statistical analyses were conducted using the SPSS 13.0 statistical software package for Windows (SPSS, Inc., Chicago, IL).

## Results

Descriptive statistics of plausible and underreporters, including anthropometric, behavioural and dietary characteristics are presented in Table 1. The underreporters were significantly younger than the plausible reporters in both genders. Compared to plausible reporters, underreporters had significantly higher values for obesity characteristics i.e. BMI, WC and sum of 6 skinfolds. Moreover, the percentage of overweight and obese individuals was significantly higher in underreporters than in plausible reporters in both genders. Among male underreporters, the percentage of subjects with high risk abdominal obesity was also higher than in plausible reporters. Male underreporters spent significantly less time in health related sports and had a lower PAL compared to male plausible reporters. In women no significant difference was observed for Tsport and PAL between plausible and underreporters. Concerning educational level and smoking status no significant difference was noticed between plausible and underreporters in both genders. As expected, underreporters reported significantly lower intake compared to plausible reporters for all macro- and micronutrients. Male underreporters had significantly higher protein, but lower fat intake when expressed as percentage of energy intake. Female underreporters reported significantly higher percentages of energy from protein, carbohydrate and fibre, and lower percentage of energy intake from fat.

**Table 1:** Anthropometric, behavioural and dietary characteristics of plausible and underreporters

	**Men**		**Women**	
		
	**Plausible**	**Underreport**	**Plausible**	**Underreport**
	n = 485	n = 382	n = 362	n = 291
Age (y)	50.2 ± 13.3	47.2 ± 13.6**	51.4 ± 12.9	44.2 ± 12.3***
BMI (kg/m^2^)	25.4 ± 3.1	26.6 ± 3.3***	24.1 ± 3.5	25.6 ± 4.4***
WC (cm)	89.5 ± 9.6	92.5 ± 10.5***	78.2 ± 9.4	80.5 ± 10.7**
Sum6SKF (mm)	76.0 ± 29.2	90.3 ± 31.7***	124.6 ± 38.6	139.5 ± 42.5***
Tsports (h/week)	3.8 ± 4.5	3.2 ± 3.7*	2.3 ± 3.2	1.9 ± 2.5
PAL (MET)	1.8 ± 0.3	1.7 ± 0.2*	1.7 ± 0.18	1.7 ± 0.15
Ttv (h/week)	14.9 ± 8.2	15.3 ± 9.1	14.6 ± 8.8	13.4 ± 8.6
BMI groups (%)		*P *< 0.001		*P *< 0.001
< 25 kg/m^2^	46.4%	33.8%	65.5%	52.6%
25–30 kg/m^2^	46.6%	51.3%	29.6%	31.6%
≥30 kg/m^2^	7.0%	14.9%	5.0%	15.8%
WC groups (%)		*P *< 0.001		N.S
< 94 or < 80 cm	68.7%	54.7%	61.3%	56.0%
94–102 or 80–88 cm	22.5%	28.8%	24.6%	23.0%
≥102 or 88 cm	8.9%	16.5%	14.1%	21.0%
Education (%)		N.S		N.S
Low	4.9%	4.5%	6.1%	5.8%
Moderate	44.5%	38.0%	42.0%	36.8%
High	50.5%	57.6%	51.9%	57.4%
Smoking (%)		N.S		N.S
Never	57.8%	56.8%	72.6%	71.7%
Former	29.5%	29.3%	15.8%	13.0%
Current	12.7%	13.9%	11.6%	15.2%
Ca (mg/d)	990.6 ± 524.7	692.9 ± 318.2***	870.3 ± 339.4	688.4 ± 324.9***
Fe (mg/d)	16.1 ± 4.7	12.0 ± 3.7***	13.3 ± 5.1	10.1 ± 3.4***
Total EI (kcal/d)	2782.0 ± 431.5	1916.1 ± 348.9***	2171.1 ± 348.1	1549.5 ± 279.4***
Protein				
(kcal/d)	425.0 ± 103.9	320.3 ± 78.9***	345.9 ± 78.8	268.2 ± 78.4***
(%E)	15.3 ± 3.0	16.8 ± 3.4***	16.0 ± 3.0	17.5 ± 5.2***
Carbohydrate				
(kcal/d)	1282.7 ± 286.5	873.3 ± 206.1***	961.2 ± 199.4	716.9 ± 172.2***
(%E)	46.1 ± 7.6	45.8 ± 7.8	44.4 ± 6.8	46.4 ± 8.1**
Sugar (kcal/d)				
(kcal/d)	293.5 ± 167.5	187.7 ± 109.9***	199.3 ± 103.6	163.7 ± 93.9***
(%E)	10.5 ± 5.6	9.9 ± 5.7	9.3 ± 4.8	10.5 ± 6.5**
Starch				
(kcal/d)	675.4 ± 207.9	469.9 ± 144.6***	486.3 ± 142.3	362.3 ± 110.5***
(%E)	24.3 ± 6.5	24.6 ± 6.7	22.5 ± 5.7	23.6 ± 6.5*
Fibre				
(kcal/d)	91.0 ± 28.9	64.9 ± 20.8***	74.3 ± 21.1	56.1 ± 17.7***
(%E)	3.3 ± 1.0	3.4 ± 1.1	3.5 ± 1.0	3.7 ± 1.2*
Fat				
(kcal/d)	951.2 ± 268.0	628.5 ± 194.9***	779.3 ± 212.3	519.8 ± 176.3***
(%E)	34.0 ± 6.8	32.4 ± 7.1**	35.6 ± 6.6	33.1 ± 8.3***
Saturated fat				
(kcal/d)	364.2 ± 114.3	240.5 ± 82.0***	265.2 ± 109.4	296.2 ± 199.5***
(%E)	13.0 ± 3.2	12.4 ± 3.2**	13.5 ± 3.1	12.7 ± 3.6**
Monounsaturated				
(kcal/d)	361.4 ± 119.5	237.9 ± 83.9***	294.4 ± 100.2	196.4 ± 79.6***
(%E)	12.9 ± 3.3	12.3 ± 3.4**	13.4 ± 3.6	12.5 ± 4.2**
Polyunsaturated				
(kcal/d)	161.0 ± 71.4	108.9 ± 50.6***	130.2 ± 65.1	86.3 ± 44.3***
(%E)	5.7 ± 2.2	5.6 ± 2.3	6.0 ± 2.8	5.5 ± 2.5*
Alcohol				
(kcal/d)	123.2 ± 125.8	93.91 ± 105.122***	84.7 ± 106.0	44.7 ± 67.1***
(%E)	4.6 ± 4.7	5.0 ± 5.7	3.9 ± 4.8	2.9 ± 4.5**
Ca (mg/d)	990.6 ± 524.7	692.9 ± 318.2***	870.3 ± 339.4	688.4 ± 324.9***
Fe (mg/d)	16.1 ± 4.7	12.0 ± 3.7***	13.3 ± 5.1	10.1 ± 3.4***
Cholesterol (mg)	104.3 ± 39.0	106.8 ± 45.8	105.3 ± 39.2	109.3 ± 46.1

* Underreporters differ significantly (*P *< 0.05) from plausible reporters of the same sex

** Underreporters differ significantly (*P *< 0.01) from plausible reporters of the same sex

*** Underreporters differ significantly (*P *< 0.001) from plausible reporters of the same sex

The differences in plausible self-reported dietary intake in men between both BMI and WC groups are presented in Table 2. Overweight and obese men had significantly higher protein, fat, cholesterol and total energy intake, expressed as kcal/day, compared to their normal weight counterparts. Overweight and obese men reported significantly less energy intake from carbohydrate, starch and fibre, but more from saturated and monounsaturated fat. These observations were confirmed by the WC classification. In addition, a significantly higher alcohol intake expressed as both kcal/day and percentage of energy intake, and significantly higher iron intake were found in men with abdominal obesity.

**Table 2:** Differences in plausible dietary intake between BMI and WC groups in men

	**BMI**				**WC**			
		
	< 25 kg/m^2^	25–30 kg/m^2^	≥30 kg/m^2^	*P*	< 94 cm	94–102 cm	≥102 cm	*P*
	n = 225	n = 226	n = 34		n = 333	n = 109	n = 43	
Total EI (kcal/d)	2758.2 ± 401.0	2777.0 ± 459.1^a^	2938.9 ± 409.6^a,b^	**<0.001**	2757.0 ± 410.8	2789.2 ± 480.8^a^	2931.0 ± 430.8^a,b^	**<0.001**
Protein								
(kcal/d)	413.0 ± 113.1	429.2 ± 88.3^a^	477.2 ± 120.5^a,b^	**<0.001**	414.4 ± 105.8	436.7 ± 95.4^a^	477.6 ± 92.1^a,b^	**<0.001**
(%E)	15.0 ± 3.2	15.5 ± 2.6	16.4 ± 4.2	0.059	15.1 ± 3.0	15.8 ± 3.0	16.4 ± 2.8^a^	**0.043**
Carbohydrate								
(kcal/d)	1326.0 ± 288.2	1242.8 ± 275.5	1260.6 ± 313.5	0.883	1308.0 ± 284.9	1232.6 ± 274.4	1213.5 ± 306.9	0.991
(%E)	48.0 ± 7.5	44.8 ± 7.3^a^	42.5 ± 6.7^a^	**<0.001**	47.4 ± 7.7	44.1 ± 6.1^a^	41.1 ± 6.6^a,b^	**<0.001**
Sugar								
(kcal/d)	310.5 ± 185.9	277.1 ± 143.4	289.7 ± 182.1	0.945	299.8 ± 174.5	284.2 ± 142.4	268.6 ± 171.3	0.408
(%E)	11.2 ± 6.2	10.0 ± 4.7	9.7 ± 5.7	0.683	10.8 ± 5.8	10.1 ± 4.7	9.0 ± 5.3	0.715
Starch								
(kcal/d)	701.3 ± 223.3	651.3 ± 186.9	663.2 ± 220.0	0.607	689.0 ± 210.8	643.9 ± 192.7	649.6 ± 215.5	0.839
(%E)	25.4 ± 7.1	23.5 ± 5.9^a^	22.3 ± 5.5^a^	**0.003**	25.0 ± 6.9	23.0 ± 5.2^a^	21.9 ± 5.4^a^	**<0.001**
Fibre								
(kcal/d)	94.4 ± 33.3	87.8 ± 24.3	89.4 ± 23.6	0.053	92.8 ± 30.6	87.0 ± 25.0	86.6 ± 23.5	0.114
(%E)	3.4 ± 1.1	3.2 ± 0.9^a^	3.1 ± 0.9^a^	**<0.001**	3.4 ± 1.0	3.2 ± 0.9^a^	3.0 ± 0.8^a^	**<0.001**
Fat								
(kcal/d)	912.0 ± 246.6	972.9 ± 283.3^a^	1066.2 ± 256.9^a^	**<0.001**	930.6 ± 260.9	979.8 ± 279.0^a^	1038.1 ± 276.6^a^	**<0.001**
(%E)	32.9 ± 6.9	34.7 ± 6.5^a^	36.2 ± 6.8^a^	**<0.001**	33.5 ± 6.9	34.8 ± 6.3^a^	35.2 ± 6.8^a^	**0.021**
Saturated fat								
(kcal/d)	347.5 ± 112.6	374.0 ± 114.5^a^	409.3 ± 107.0^a^	**<0.001**	355.9 ± 113.5	373.2 ± 112.9^a^	405.9 ± 115.0^a^	**<0.001**
(%E)	12.6 ± 3.4	13.4 ± 3.0^a^	13.9 ± 3.0^a^	**0.006**	12.8 ± 3.3	13.3 ± 3.0	13.8 ± 3.2	0.111
Monounsaturated								
(kcal/d)	343.1 ± 114.3	371.3 ± 122.3^a^	417.6 ± 111.4^a,b^	**<0.001**	352.8 ± 121.0	373.2 ± 111.4^a^	398.7 ± 120.5^a^	**<0.001**
(%E)	12.4 ± 3.5	13.2 ± 3.1^a^	14.2 ± 3.5^a^	**<0.001**	12.7 ± 3.5	13.3 ± 2.9^a^	13.5 ± 3.5^a^	**0.025**
Polyunsaturated								
(kcal/d)	153.5 ± 65.3	165.9 ± 76.3^a^	178.4 ± 72.7^a^	**0.001**	156.3 ± 66.2	169.6 ± 86.2^a^	176.3 ± 65.8^a^	**<0.001**
(%E)	5.6 ± 2.2	5.9 ± 2.3	6.0 ± 2.1	0.072	5.6 ± 2.1	6.0 ± 2.5	6.0 ± 1.9	0.105
Alcohol								
(kcal/d)	109.6 ± 114.8	134.6 ± 133.4	137.6 ± 138.0	0.571	106.4 ± 111.7	142.5 ± 130.6^a^	204.4 ± 172.9^a,b^	**<0.001**
(%E)	4.1 ± 4.3	5.0 ± 5.0	5.0 ± 5.1	0.877	4.0 ± 4.2	5.3 ± 4.8	7.4 ± 6.3^a,b^	**0.003**
Ca (mg/d)	990.2 ± 643.6	980.2 ± 394.5	1036.2 ± 397.7	0.408	970.2 ± 567.9	1021.5 ± 402.5	1070.8 ± 440.0	0.099
Fe (mg/d)	15.9 ± 4.4	16.0 ± 4.93	17.6 ± 5.4	0.068	15.8 ± 4.2	16.2 ± 5.9	17.6 ± 5.1^a^	**0.030**
Cholesterol	99.4 ± 38.4	106.9 ± 39.3	119.1 ± 36.4^a^	**0.030**	101.2 ± 39.8	105.4 ± 35.3	124.7 ± 36.0^a,b^	**0.007**

Results of ANCOVA's with age, time health related sports and time watching TV as covariatesa

^a ^*P *< 0.05 vs. BMI < 25 kg/m^2^

^b ^*P *< 0.05 vs. BMI 25–30 kg/m^2^

Table 3 gives an overview of the difference in plausible dietary intake between BMI and WC groups in women. Significantly higher values for carbohydrate, starch, fibre, fat (saturated, mono and polyunsaturated) and total energy intake were observed in overweight and obese women compared to normal weight women. Obese women also reported significantly higher intake of protein, sugar and iron intake than their normal weight counterparts. Similar findings were observed between the WC groups. Women with high risk abdominal obesity reported a significantly higher cholesterol intake and higher energy intake from alcohol compared to women with normal WC or moderate risk for abdominal obesity.

**Table 3:** Differences in plausible dietary intake between BMI and WC groups in women

	**BMI**				**WC**			
		
	< 25 kg/m^2^	25–30 kg/m^2^	≥30 kg/m^2^	*P*	< 80 cm	80–88 cm	≥88 cm	*P*
	n = 237	n = 107	n = 18		N = 222	n = 89	n = 51	
Total EI (kcal/d)	2151.1 ± 340.9	2176.0 ± 341.3^a^	2369.8 ± 425.4^a,b^	**<0.001**	2145.9 ± 340.2	2177.8 ± 330.1^a^	2255.9 ± 399.7^a,b^	**<0.001**
Protein								
(kcal/d)	341.9 ± 81.0	348.8 ± 67.0	381.3 ± 104.7^a^	**0.017**	339.7 ± 83.3	353.1 ± 63.9^a^	360.3 ± 80.5^a^	**0.003**
(%E)	16.0 ± 3.2	16.1 ± 2.7	16.0 ± 2.6	0.616	15.9 ± 3.2	16.3 ± 2.7	16.0 ± 2.5	0.381
Carbohydrate								
(kcal/d)	952.0 ± 188.8	968.3 ± 217.6^a^	1040.6 ± 215.2^a^	**<0.001**	950.9 ± 193.4	971.6 ± 188.5^a^	987.7 ± 240.6^a^	**<0.001**
(%E)	44.4 ± 6.6	44.5 ± 7.3	44.1 ± 6.5	0.952	44.4 ± 6.7	44.7 ± 6.4	43.9 ± 7.9	0.691
Sugar								
(kcal/d)	197.5 ± 96.9	197.7 ± 115.3	233.3 ± 116.0^a^	**0.045**	198.8 ± 93.1	197.2 ± 118.1	205.0 ± 120.7	0.076
(%E)	9.3 ± 4.5	9.1 ± 5.5	9.7 ± 4.1	0.803	9.4 ± 4.3	9.1 ± 5.5	9.1 ± 5.6	0.968
Starch								
(kcal/d)	479.6 ± 125.0	489.2 ± 171.5^a^	557.8 ± 152.7^a,b^	**0.002**	483.2 ± 133.7	479.8 ± 148.3	511.3 ± 166.5^a,b^	**0.004**
(%E)	22.5 ± 5.3	22.4 ± 6.4	23.7 ± 5.9	0.637	22.6 ± 5.3	22.1 ± 6.1	22.6 ± 6.0	0.616
Fibre								
(kcal/d)	71.7 ± 19.0	78.3 ± 23.4^a^	83.5 ± 27.1^a^	**0.021**	71.5 ± 19.5	78.5 ± 20.9	79.0 ± 26.0^a^	**0.048**
(%E)	3.4 ± 0.9	3.6 ± 1.0	3.6 ± 1.0	0.910	3.4 ± 0.9	3.7 ± 1.0	3.5 ± 0.9	0.133
Fat								
(kcal/d)	776.9 ± 210.7	770.9 ± 214.2^a^	860.2 ± 217.3^a,b^	**<0.001**	782.6 ± 209.4	759.8 ± 206.5^a^	799.1 ± 235.5^a,b^	**<0.001**
(%E)	35.8 ± 6.6	35.1 ± 6.8	36.3 ± 6.4	0.250	36.2 ± 6.6	34.6 ± 6.3	35.1 ± 7.1	0.485
Saturated fat								
(kcal/d)	295.3 ± 87.4	294.9 ± 93.0^a^	314.5 ± 95.0^a^	**0.006**	295.8 ± 87.2	289.7 ± 91.8^a^	308.9 ± 94.2^a,b^	**<0.001**
(%E)	13.6 ± 3.0	13.4 ± 3.2	13.2 ± 3.3	0.622	13.7 ± 3.0	13.2 ± 3.1	13.6 ± 3.2	0.313
Monounsaturated								
(kcal/d)	291.3 ± 99.9	296.0 ± 101.4^a^	326.2 ± 98.2^a^	**<0.001**	294.3 ± 100.6	289.6 ± 96.3^a^	303.3 ± 106.5^a^	**0.001**
(%E)	13.4 ± 3.6	13.5 ± 3.8	13.7 ± 3.3	0.203	13.6 ± 3.6	13.2 ± 3.5	13.3 ± 3.7	0.632
Polyunsaturated								
(kcal/d)	127.4 ± 67.3	130.6 ± 55.5^a^	165.2 ± 79.3^a,b^	**<0.001**	129.6 ± 67.3	126.0 ± 54.4	140.0 ± 71.9^a^	**0.010**
(%E)	5.9 ± 3.0	5.9 ± 2.1	7.2 ± 3.6	0.073	6.0 ± 3.0	5.7 ± 2.2	6.1 ± 2.9	0.513
Alcohol								
(kcal/d)	82.1 ± 102.9	90.0 ± 115.1	89.8 ± 93.1	0.821	74.6 ± 98.5	95.1 ± 108.6	110.8 ± 127.1	0.563
(%E)	3.8 ± 4.7	4.2 ± 5.4	3.6 ± 3.5	0.501	3.5 ± 4.6	4.4 ± 4.7	5.0 ± 5.8	0.888
Ca (mg/d)	855.6 ± 303.0	884.2 ± 351.2	980.8 ± 620.8	0.142	846.8 ± 308.5	890.7 ± 348.8	936.6 ± 435.2^a^	**0.041**
Fe (mg/d)	13.0 ± 3.5	13.3 ± 4.1	17.8 ± 15.5^a,b^	**<0.001**	13.0 ± 3.4	13.2 ± 4.2	14.8 ± 9.9^a^	**0.030**
Cholesterol	104.2 ± 38.6	110.1 ± 41.1	91.3 ± 31.6	0.138	105.1 ± 40.3	105.8 ± 39.4	105.0 ± 34.0	0.353

Results of ANCOVA's with age, time health related sports, time watching TV as covariatesa

^a ^*P *< 0.05 vs. BMI < 25 kg/m^2^

^b ^*P *< 0.05 vs. BMI 25–30 kg/m^2^

Differences in dietary intake between subjects not participating in health related sports, participating less than 3.5 h/week and 3.5 h/week or more in health related sports are presented in Table 4. Men participating in health related sports for at least 3.5 h/week showed significantly lower values for fat, saturated and monounsaturated fat, expressed as both kcal/day and percentage of total energy intake compared to sedentary men. A similar finding concerning fat intake was also observed in women, however, not significant. On the other hand, women participating in sports 3.5 h/week or more reported significantly higher intake of carbohydrates and sugars compared to women less involved in sport activities. No significant difference was observed for BMI between men and women with different levels of sports participation. On the other hand, men participating in health related sports 3.5 h/week or more had significantly lower values for the sum of 6 skinfolds.

**Table 4:** Differences in plausible dietary intake in men and women with different levels of sports participation

	**BMI**				**WC**			
		
**Tsports**	0 h/week	< 3.5 h/week	≥3.5 h/week	*P*	0 h/week	< 3.5 h/week	≥ 3.5 h/week	*P*
	n = 129	n = 157	n = 199		n = 123	n = 159	n = 80	
Total EI (kcal/d)	2729.3 ± 439.3	2831.4 ± 433.4	2777.3 ± 422.6	0.687	2139.9 ± 339.6	2199.7 ± 342.5	2162.1 ± 371.1	0.805
Protein								
(kcal/d)	411.7 ± 93.1	429.8 ± 91.7	429.9 ± 118.4	0.217	337.7 ± 78.6	354.7 ± 82.3	340.8 ± 70.5	0.182
(%E)	15.1 ± 2.7	15.3 ± 2.8	15.5 ± 3.4	0.181	15.8 ± 3.0	16.2 ± 3.2	15.9 ± 2.6	0.119
Carbohydrate								
(kcal/d)	1224.2 ± 292.3	1309.4 ± 275.7	1299.4 ± 287.2	0.303	946.6 ± 198.9	951.1 ± 1206.2	1003.8 ± 182.2^a,b^	**0.019**
(%E)	44.9 ± 8.5	46.4 ± 7.3	46.7 ± 7.1	0.408	44.3 ± 6.6	43.3 ± 6.7	46.8 ± 6.7^a,b^	**< 0.001**
Sugar								
(kcal/d)	272.1 ± 156.9	293.0 ± 169.5	307.8 ± 171.9	0.452	192.5 ± 111.5	192.8 ± 99.2	222.7 ± 97.4^a,b^	**0.027**
(%E)	9.9 ± 5.3	10.3 ± 5.5	11.0 ± 5.7	0.337	9.1 ± 5.5	8.8 ± 4.2	10.4 ± 4.5^b^	**0.026**
Starch								
(kcal/d)	649.9 ± 190.2	685.2 ± 220.3	684.1 ± 208.3	0.656	484.6 ± 148.0	483.3 ± 140.3	495.1 ± 138.5	0.469
(%E)	23.8 ± 5.7	24.3 ± 6.9	24.7 ± 6.8	0.710	22.6 ± 5.4	22.1 ± 5.8	23.1 ± 6.0	0.455
Fibre								
(kcal/d)	87.0 ± 30.6	92.1 ± 26.9	92.6 ± 29.1	0.211	75.0 ± 21.9	71.8 ± 20.7	78.0 ± 20.2	0.220
(%E)	3.2 ± 1.1	3.3 ± 1.0	3.3 ± 0.9	0.295	3.5 ± 0.9	3.3 ± 0.9	3.7 ± 1.1	0.088
Fat								
(kcal/d)	978.8 ± 285.5	972.3 ± 276.3	916.6 ± 246.4^a^	**0.032**	769.9 ± 189.4	804.2 ± 219.5	744.2 ± 227.0	0.529
(%E)	35.6 ± 7.4	34.0 ± 6.7^a^	32.8 ± 6.2^a^	**< 0.001**	35.9 ± 6.3	36.2 ± 6.6	34.0 ± 7.0	0.100
Saturated fat								
(kcal/d)	375.8 ± 115.3	376.0 ± 120.8	347.4 ± 106.5^a^	**0.028**	292.4 ± 78.5	303.9 ± 91.5	286.6 ± 99.7	0.857
(%E)	13.7 ± 3.3	13.2 ± 3.3	12.4 ± 3.0^a,b^	**0.003**	13.6 ± 2.7	13.7 ± 3.2	13.0 ± 3.3	0.400
Monounsaturated								
(kcal/d)	379.8 ± 129.4	365.1 ± 125.1^a^	346.6 ± 106.2^a^	**0.012**	289.2 ± 86.0	306.8 ± 107.2	278.0 ± 104.4	0.338
(%E)	13.8 ± 3.6	12.8 ± 3.4^a^	12.4 ± 3.1^a^	**< 0.001**	13.5 ± 3.3	13.8 ± 3.7	12.7 ± 3.7	0.148
Polyunsaturated								
(kcal/d)	166.9 ± 79.7	159.7 ± 70.5	158.3 ± 66.4	0.239	128.3 ± 75.2	134.6 ± 60.2	124.2 ± 57.2	0.807
(%E)	6.0 ± 2.5	5.6 ± 2.2	5.7 ± 2.1	0.137	6.0 ± 3.6	6.0 ± 2.3	5.7 ± 2.3	0.694
Alcohol								
(kcal/d)	114.6 ± 124.1	119.8 ± 131.4	131.4 ± 122.5	0.112	85.6 ± 102.4	89.7 ± 115.3	73.4 ± 91.4	0.232
(%E)	4.3 ± 4.7	4.3 ± 4.7	4.9 ± 4.7	0.100	4.0 ± 4.5	4.2 ± 5.4	3.3 ± 4.1	0.121
Ca (mg/d)	929.5 ± 348.1	1024.5 ± 431.4	1003.5 ± 666.7	0.326	854.3 ± 356.7	882.7 ± 332.8	870.2 ± 328.1	0.738
Fe (mg/d)	15.3 ± 4.1	16.5 ± 5.4	16.1 ± 4.5	0.068	13.5 ± 7.0	13.1 ± 3.7	13.3 ± 3.8	0.879
Cholesterol	107.5 ± 33.7	104.3 ± 40.2	102.1 ± 41.3	0.803	105.9 ± 38.3	102.0 ± 38.4	110.8 ± 41.7	0.463
*BMI	26.0 ± 2.9	25.4 ± 3.2	25.0 ± 3.2	0.141	24.8 ± 4.0	23.5 ± 3.1	24.3 ± 3.2	0.234
*Sum 6 SKF	82.6 ± 28.5	81.5 ± 31.9	67.3 ± 25.11^a,b^	**< 0.001**	128.1 ± 37.4	124.1 ± 38.6	120.7 ± 40.2	0.252

Results of ANCOVA's with age and BMI covariates

*Results of ANCOVA's with age as covariate

^a ^P < 0.05 vs. Tsports = 0 h/week

^b ^P < 0.05 vs. Tsports < 3.5 h/week

## Discussion

Although the role of dietary intake, especially the role of fat intake, in the development of obesity has been widely discussed in the literature, uncertainties remain because of conflicting results reported by cross-sectional and prospective studies [37]. This could be partially explained by the phenomenon of underreporting when using self-reported dietary intake. Most studies in nutritional epidemiology mention underreporting as an important bias. If underreporters are not excluded, spurious associations between dietary intake and obesity may be found. However, only few studies identified and excluded underreporters [4,7,38].

In the present study, plausible reporters were compared to underreporters for a number of anthropometric, behavioural and dietary characteristics. Underreporting is commonly associated with obesity quantified by body mass index [8,12-15,39]. In this study, a significantly larger percentage of overweight, obese, but also abdominally obese individuals were observed among underreporters. Underreporters were also significantly younger, but no significant difference was found for educational level and smoking status between plausible and underreporters. Some studies found underreporting to be more prevalent among older [13,14], less [40] or more educated [14] subjects and smokers [13]. Again other studies reported, similarly to our results, a higher proportion of underreporting among younger subjects [39,41] and no significant difference for educational level between under- and plausible reporters [13,38]. In the present study, male underreporters participated significantly less in health related sports and had lower PAL compared to plausible reporters. These results are in agreement with the study of Johansson et al. [14] indicating lower activity scores among underreporters. In our study, total daily energy intake and intake of all macronutrients were lower among underreporters than among accurate reporters, and underreporters reported a significantly higher percentage of energy from protein, but lower percentage from fat. These results are in accordance with the literature indicating that underreporters and obese persons tend to underreport foods rich in fat and sugar, and overreport protein intake [12,14,38]. Nevertheless, carbohydrates expressed as percentage of energy were significantly higher among female underreporters compared to their plausible counterparts in the present study. A number of the aforementioned inconsistent results may be caused by different dietary assessment methods (FFQ vs. diet diary) and different methods used to identify implausible dietary intake (i.e. doubly labelled water, biomarkers, ratio of reported energy intake to basal metabolic rate). Differences in age, culture and health consciousness among the populations studied may also be responsible for these inconsistencies.

The main purpose of the present study was to analyse the associations of dietary intake with BMI and WC after excluding the underreporters. A first important finding was that fat intake (kcal/day), including saturated, mono- and polyunsaturated fat, was significantly higher in overweight and (abdominally) obese subjects compared to their normal weight counterparts in both genders, statistically controlling age and physical activity variables. In addition, percentage of energy intake from fat was significantly higher in overweight, obese and abdominally obese men compared to men with normal weight or WC. On the other hand, this observation was not significant in women. In the literature, the role of dietary fat in the development of obesity and abdominal obesity is equivocal. Some cross-sectional studies found no association [17,42], while other observed a positive association [16,18] between higher fat intake and obesity. In the study of Garaulet et al. it is suggested that, even though obesity is a multifactorial phenomenon, dietary intake, especially fat intake, is the most important factor contributing to obesity [43]. Larson et al. [44] indicated that dietary fat plays a minor role in increasing overall body fat but not specifically influences fat increase in the intra-abdominal region. In a prospective study of Koh-Banerjee et al. [26] a 2% increment in energy intake from unsaturated (trans) fat resulted in a 0.77 cm waist gain over 9 year, whereas in other prospective studies no association was found between intake of dietary fat and abdominal obesity [24,25,45]. A possible explanation for the lack of association in these latter studies could be that implausible reporters of dietary intake were not always excluded. Nevertheless, the results of the present study confirm the relationship between fat intake and both BMI and WC. Several arguments have been proposed for this association. Firstly, fat is the most energy dense macronutrient. Secondly, fat provides a lower satiety feeling and its great flavour and palatability may lead to a greater consumption of fatty foods. Authors also reported that fat has a lower thermogenic effect than carbohydrates and proteins [46,47], which results in lower energy expenditure and consequently in larger fat stores. Finally, diet currently contains a lot of added sugars and fats as the food industry has made added sugars and vegetable oils accessible at a remarkably low cost [2].

An important asset of the present study is that besides fat intake, the association of other macronutrients and total energy intake with obesity has also been analysed. According to the BMI and WC classification in both men and women, total energy intake was found to be positively associated with obesity. Slattery et al. [42] found a positive correlation of total energy intake with waist-hip-ratio, but not with BMI in white women, but they found no significant correlation in men. In contrast, in a study of Trichopoulou et al. [48], higher energy intake was significantly associated with waist-hip-ratio independently of BMI in men but not in women. In other studies, there was no significant difference in energy consumption among the BMI categories [17,18]. Following our results controlled for age, Tsports and Ttv, energy percentages from sugars, starch and fibres are negatively related to BMI and WC in men, whereas in women belonging to the overweight or obesity category is associated with higher carbohydrate and fibre intake (kcal/day). In the literature, overweight and obese individuals are reported to consume generally less carbohydrates [16-18,42] and dietary fibres [4,18,26,42,49] than their normal weight counterparts. Several physiological mechanisms for the role of fibre on weight regulation are proposed in the literature. The energy content of fibre per unit weight food is low. Consequently, inclusion of fibre in a diet reduces energy density. Dietary fibre tends to reduce dietary intake by slowing digestion and absorption of nutrients, and by increasing the production of gut hormones enhancing satiety feeling. Moreover, some types of fibre reduce the overall absorption of fat and protein [5]. In the present study, regarding carbohydrate, starch, sugar and fibre intake, opposite results were found for men and women. In men, energy percentages from carbohydrates, starch and fibres are significantly lower in overweight and obese men compared to lean men, whereas in women the intake of carbohydrates, starch, sugars and fibres were found to be positively related with overweight and obesity. A possible explanation for this sex difference regarding fibre intake could be that women tend to be more health-conscious and more knowledgeable about food and nutrition than men [50-52]. In addition, one could assume that fibre intake will generally increase with higher total energy intake which is the case in overweight and obese women, although this is not the case in men.

As to protein intake, the positive association with (abdominal) obesity in both genders is in agreement with the findings of Slattery et al. [42]. Conversely, Davis et al. [18] reported that protein intake did not differ between BMI groups. Relationships found between alcohol consumption and body weight or fat distribution are inconsistent and seem to be sex specific [53]. In the present study, men and women with high risk abdominal obesity reported a significantly higher percentage of energy intake from alcohol. These results support the findings of other investigators that moderate to heavy drinkers of both genders have a larger WC or higher waist-hip-ratio than light drinkers [42,53,54]. However, when using the BMI classification only, no difference was found in alcohol intake between the groups. In other studies, BMI was found to be negatively related to alcohol intake in women, whereas a slight positive association was observed in men [53,55,56]. Studies on the association of obesity with iron and cholesterol intake are sparse. Our results show a significantly higher consumption of iron and cholesterol among men and women with abdominal obesity. These results might partially be explained by the higher fat and protein intake among obese individuals in our sample.

The findings of the present study revealed that regarding dietary intake some sex differences were observed between obese men and women. Obese women show a higher consumption of all macronutrients, and surprisingly also of fibres, while men show a higher fat and protein intake, but a lower intake of carbohydrates and fibres. In addition, there was a positive association between alcohol intake and abdominal obesity in men, but not in women. On the other hand, women with abdominal obesity show a significantly higher calcium intake compared to lean women, but this trend was not significant in men.

Another purpose of this study was to investigate whether plausible dietary intake varies between subjects with different level of sports participation after adjustment for age and BMI. Subjects participating in health related sports reported higher intake of carbohydrates, but lower intake of fat compared to subjects not participating in sport activities. An explanation of this finding can be that individuals participating in health related sports are more health conscious and are more prone to adopt a low fat – high carbohydrate diet than sedentary individuals less well concerned with health. It is well accepted that carbohydrates are one of the most important components in sports nutrition. In addition, there is evidence that dietary intake can be influenced by physical activity. High intensity exercise induces a suppression of appetite, and long duration, high-intensity exercise has a stronger effect than a short duration exercise period [57]. The higher sugar intake in physically active women might be explained by the assumption that these women try to compensate their higher intake of sweets by doing more sports.

Several limitations should be considered when interpreting results of the present study. The first limitation is the cross-sectional design, which inhibits to establish causal relationships as both the effect (obesity) and the potential causes (dietary intake and other factors) are measured simultaneously. Second, the use of self-reported data for dietary intake and physical activity depends for a large part on the cooperation and honesty of the participants. On the other hand, the 3-day diet diary [28] and the physical activity questionnaire (FPACQ) [31] used in our study have been properly validated previously. Although these instruments have limitations, they are easy to administer and low in cost, whereas the use of more objective methods, such as doubly labelled water technique, indirect calorimetry or accelerometers is unfeasible in large scale epidemiological studies. Another limitation is related to the representativeness of the sample. Although the original sample was randomly selected by the National Institute of Statistics, our sample consisted of volunteers, presumably being more health conscious persons. Moreover, the exclusion of the implausible reporters resulted in a reduction of the sample size and a more select sample which could be considered as a potential weakness. However, excluding the implausible reporters increased the validity of the dietary reports of the subjects and permitted to avoid spurious conclusions. When the analyses were performed on the total sample, associations of obesity indicators with dietary intake, among others total energy and fat intake, were not observed. This finding reinforces the importance of identifying and excluding implausible reporters. A major strength of our study is that dietary intake was not restricted to fat intake but included a large number of dietary factors (total energy intake, macronutrients expressed as both kcal/day and percentage from total energy intake, alcohol, calcium, iron and cholesterol) and that the analyses were adjusted for age and physical activity variables. Another strength is that WC, an indicator of abdominal obesity was also used besides BMI. Finally, it is important to mention that the anthropometric data were not self-reported, but measured by trained staff.

## Conclusion

The present study highlighted the importance of excluding implausible dietary reports when investigating associations of overweight and obesity with dietary intake. If underreporters are not excluded, one runs the risk that no or other erroneous conclusions are drawn. Bearing in mind the limitations related to a cross-sectional design, implying that no cause-effect relationship could be drawn, the findings of the present study seem to support that dietary intake might play a role in the development of overweight and abdominal obesity. Overweight and obese men reported a higher consumption of fats and proteins, whereas their energy percentages from carbohydrates and fibres were lower compared to their normal weight counterparts. On the other hand, in overweight and obese women, a higher intake of all macronutrients was observed compared to lean women. The positive association between alcohol intake and abdominal obesity was confirmed by the present results in men, but not in women. The sex differences for dietary intake between obese men and women might reflect the generally higher health consciousness of women. In addition, the current findings pointed to sex differences between men and women with high level of sports participation. The present study also highlighted the importance of waist circumference as additional measurement in epidemiological studies, as alcohol and calcium intake are only associated with waist circumference, and not with BMI. Besides enhancing sports and physical activity participation, it is necessary to improve the knowledge about nutrition among the Flemish population and to promote the well-balanced consumption of wholesome food.

## Competing interests

The author(s) declare that they have no competing interests.

## Authors' contributions

ND, KW and LM participated in the data collection. WD, JL, RP and MT helped with the study design. ND analysed the data and wrote a first version of the manuscript. All authors provided comments on the drafts and assisted in editing the manuscript. They all read and approved the final version of the manuscript.
